# Investigating the relationship between consultation length and quality of tele-dermatology E-consults in China: a cross-sectional standardized patient study

**DOI:** 10.1186/s12913-022-08566-2

**Published:** 2022-09-22

**Authors:** Xue Gong, Mengchi Hou, Rui Guo, Xing Lin Feng

**Affiliations:** 1 School of Public Health, Capital Medical University, Beijing, China; 2 China Aerospace Science & Industry Corporation 731 Hospital, Beijing, China; 3 Department of Health Policy and Management, School of Public Health, Peking University, Beijing, China

**Keywords:** Consultation length, Quality of E-consults, Tele-dermatology, Standardized patients, China

## Abstract

**Background:**

Consultation length, the time a health provider spend with the patient during a consultation, is a crucial aspect of patient-physician interaction. Prior studies that assessed the relationship between consultation length and quality of care were mainly based on offline visits. Research was lacking in E-consults settings, an emerging modality for primary health care. This study aims to examine the association between consultation length and the quality of E-consults services.

**Methods:**

We defined as standardized patient script to present classic urticaria symptoms in asynchronous E-consults at tertiary public hospitals in Beijing and Hangzhou, China. We appraised consultation length using six indicators, time waiting for first response, time waiting for each response, time for consultation, total times of provider’s responses, total words of provider’s all responses, and average words of provider’s each response. We appraised E-consults services quality using five indicators building on China’s clinical guidelines (adherence to checklist; accurate diagnosis; appropriate prescription; providing lifestyle modification advice; and patient satisfaction). We performed ordinary least squares (OLS) regressions and logistic regressions to investigate the association between each indictor of consultation length and E-consults services quality.

**Results:**

Providers who responded more quickly were more likely to provide lifestyle modification advice and achieve better patient satisfaction, without compromising process, diagnosis, and prescribing quality; Providers who spent more time with patients were likely to adhere to clinical checklists; Providers with more times and words of responses were significantly more likely to adhere to the clinical checklist, provide an accurate diagnosis, appropriate prescription, and lifestyle modification advice, which achieved better satisfaction rate from the patient as well.

**Conclusions:**

The times and words that health providers provide in E-consult can serve as a proxy measure for quality of care. It is essential and urgent to establish rules to regulate the consultation length for Direct-to-consumer telemedicine to ensure adequate patient-provider interaction and improve service quality to promote digital health better.

**Supplementary Information:**

The online version contains supplementary material available at 10.1186/s12913-022-08566-2.

## Background

Average consultation length is the time that medical personnel spends with patients in the process of consultation, including history taking, treatment planning, discussing substance use, health education, and so on, which is a quality indicator of promoting safe and cost-effective use of drugs around the world as suggested by the World Health Organization (WHO) and the International Network for the Rational Use of Drugs (INRUD) [1–3]. Studies indicated that consultation length might become a proxy measure of the quality of care and influence the delivery of health care [4, 5]. The optimum WHO/INRUD value for average consultation time is ≥ 10 min, which was taken as the standard by most scholars in developing countries, for example in Pakistan [6], Kenya [7], Ethiopia [8], Kuwait [9], and so on. The Royal College of General Practitioners has recommended that primary care appointments be at least 15 min long, including examinations [10]. In addition, Egypt recommends 30 min per patient as the optimum consultation length in primary care [11]. However, in two recent systematic reviews based on WHO/INRUID patient care indicators, only two studies were consistent with WHO/INRUID recommended average consultation, and the average consultation time was 18.16 and 10.46 min, respectively [12, 13]. A review of studies involving 67 countries reported that the average consultation length ranged from 48 s in Bangladesh to 22.5 min in Sweden.

Consultation length is a crucial aspect of patient-physician interaction [14], and Irving et al. (2017) [2] found significant positive associations between longer consultation length and higher healthcare spending per capita, reduced hospital admissions for diabetes, higher primary care physician density, higher physician efficiency, and higher physician satisfaction. Research on consultation length and its impact has been conducted in a range of international primary care settings [15]. However, the impact of consultation length on health outcomes in general practice has long been debated. A systematic review by Wilson et al. (2002) [16] explored associations between consultation length, process and outcomes and found that doctors who had longer average consultation lengths prescribed less and were more likely to include lifestyle advice and preventive activities. Another review suggested that there was evidence of an improved diagnosis of psychological problems in longer consultations, and time is a significant barrier to treating depression [17]. Crucially, longer consultation lengths could also benefit physicians, including reduced burnout and improved job satisfaction [18]. While a Cochrane systematic review of clinical trials reported that there was insufficient evidence to say whether increasing consultation length provides patient benefit; several aspects of doctors’ behavior (prescribing, referral, investigation, and reconsultation) remained unchanged despite significant changes in appointment length [19, 20]. A review on the association between consultation length and patient’s perception of care concluded that it was not the length of the consultation but instead the patient's psychosocial factors that improved consultation outcome [21]. Therefore, the potential association between consultation length and quality of care needs to be further explored.

Common methods for measuring health care quality included direct clinical observations, patient exit interviews, chart abstraction, vignettes or written case simulations, standardized patients (SPs), etc. [22, 23]. The standardized patients (SPs) method is widely regarded as the “gold standard” for measuring the medical practice of providers and can avoid the "Hawthorne effect" [24–29]. Existing studies have used the SPs method to explore the relationship between consultation length and health care quality. For example, Gao et al. (2022) [30] and Goedhuys et al. (2001) [31] found that longer consultation time resulted in higher patient satisfaction; Goedhuys et al. (2001) [31] indicated longer consultations were also rated higher for the quality of the communication; Epstein et al. (2005) [32] also noted patient-centered communication is associated with increased visit length; Banerjee et al. (2020) [33] and Wang et al. (2022) [4] found that providers who spent more time with patients were significantly more likely to adherence more checklist items of recommended questions and examinations; besides, Wang et al. (2022) [4] found providers who consulted longer time with patients were more likely to give a correct diagnosis. In conclusion, the relationship between consultation length and health care quality has been reasonably well established in face-to-face interactions.

The COVID-19 crisis has presented multiple barriers to health care, including patients’ and providers’ fears of acquiring infection through travel to healthcare facilities [34]. Direct-to-consumer (DTC) telemedicine addresses the diagnosis, treatment, and monitoring of patients by means of electronic technology, which is well suited for scenarios in which infrastructure remains intact and clinicians are available to see patients online to provide synchronous and asynchronous support for patients who require routine clinical services [34–36]. Electronic consultations (E-consults), as a means of asynchronous communication between clinicians and patients, improve patient access to specialist care [37, 38]. The utility and feasibility of E-consults have been demonstrated across multiple specialties, including cardiology, gastroenterology, endocrinology, infectious disease, nephrology, and dermatology [38]. Nevertheless, no research on consultation duration and its impact has been found in the E-consults setting, so it is unclear whether the established relationship between the two in face-to-face interactions exists in the E-consults setting.

By December 2021, the number of online medical users had reached 298 million in China, accounting for 28.9 percent of the total online users [39]. With the increasing demand for E-consults with better outcomes, this study examines the association between consultation length and the quality of E-consults services to get new insights for potential policies and practices that could improve online health service quality in China.

## Methods

### Study design and data collection

This study is part of a cross-sectional standardized patient study on the quality of tele-dermatology E-consults in China (details of the original study design are described elsewhere). In brief, we focused on tertiary public hospitals in Beijing and Hangzhou that offer E-consults dermatology services and performed comprehensive internet searches for physicians from the departments of Dermatology, Allergy and Immunology, Traditional Chinese Medicine, or other related departments. We excluded physicians with live/synchronous video consultations and included all physicians with asynchronous graphic consultations of dermatology in the study. We utilized SPs to perform urticaria during the consultations because the SPs method was widely accepted as the "gold standard" in evaluating healthcare services [24, 29]. King et al. (2019) [40] proposed ten questions to consider when assessing suitability as an SP case; specific reasons for choosing urticaria are presented in the additional file 1.

Physicians and professors with rich experience were invited to develop a standard script for the SPs to use during the clinical encounter. The script covered all possible questions a physician may ask, as well as the SP’s answers during the consultation. The script included five sections: (1) a detailed background story, (2) an opening statement of symptoms portrayed by SPs (Doctor, there has been wheals all over my body with itch. Could you please figure this out for me?), (3) an illness history presented in a question-and-answer format, (4) the language to insist on diagnosis/prescription/lifestyle advice if not given and (5) an ending. Examples of specific consultations are presented in additional file 2. Each encounter was performed according to the script.

In each encounter, the provider was expected to diagnose the patient and provide medical advice. Following each consultation, the SP completed a semi-structured questionnaire to collect the details of each consultation. Finally, we calculated the valid consultation rate; that is, SP conducted research strictly according to the script, the doctor responded, and SP recorded the doctor-patient interaction process completely. The valid consultation rate was 76%, and a total of 87 doctor-patient interactions were included in the study. Specific demographic characteristics are shown in additional file 3. No physicians voiced any suspicions during the E-consults.

### Measurement of consultation length and the quality

We appraised the consultation length of tele-dermatology using six indicators (Table 1): (1) time waiting for first response, defined as the time interval between the patient’s first question and the doctor’s first response. (2) time waiting for each response, defined as the average time interval between each patient’s question and the doctor’s response. (3) time for consultation, defined as the time interval between the patient’s first question and the doctor’s last response. (4) total times of provider’s responses, defined as the total number of times of doctor’s responses in one encounter. (5) total words of provider’s all responses, defined as the total number of words replied by the doctor in one encounter. (6) average words of provider’s each response, defined as the average number of words replied by the doctor of each response in one encounter. We appraised the quality of E-consults services using five indicators (Table 1): (1) adherence to checklist. According to the Guideline for the Diagnosis and Treatment of Urticaria (2018) [41], twenty-three consultation items in 6 components (onset time and duration, main symptoms, following symptoms, trigger and regulation, medical record, and past history and family history) should be queried in the consultation. We defined adherence to checklist as the percent of items completed for clinical guideline stipulated consultation. (2) accurate diagnosis, defined as urticaria, not allergy, erythema annulare, popular urticaria, or other diseases. (3) appropriate prescription. In the script, our standardized patient was designed as being treated with an antihistamine but achieved no alleviations. It is recommended by the Guideline for the Diagnosis and Treatment of Urticaria (2018) [41] that a second-line medication, such as changing the antihistamines or using other antihistamines in combination, should be prescribed. According to the Hospital Prescription Review Management Specification (Trial) [42] in China, a standardized prescription includes definite medication, course duration, and dosage. Therefore, we defined appropriate prescription as the doctor prescribing a definite second-line medication with clearly defined course duration and dosage. (4) providing lifestyle modification advice, defined as whether the doctor provides lifestyle modification advice. (5) patient satisfaction, defined as whether SP is satisfied with the experience during the whole process of E-consults.

**Table 1:** Variables of characteristics of physicians, the consultation length and E-consults services quality of tele-dermatology

Variable name	Description	Type	Coding	Source
1. Characteristics				
1.1 Region	Doctor’s practice place	Dichotomous	0: Beijing, 1: Hangzhou	Records
1.2 Sex	Doctor’s sex	Dichotomous	0: Male, 1: Female	Records
1.3 Institutional type	Type of institution where doctor works	Dichotomous	0: Traditional Chinese medicine hospital, 1: General hospital	Records
1.4 Title	Doctor’s title (Senior: associate chief physician, chief physician; Junior: resident physician, attending physician)	Dichotomous	0: Senior,1: Junior	Records
2. Consultation length				
2.1 Time waiting for first response	The time interval between the patient’s first question and the doctor’s first response	Continuous	minutes	Records
2.2 Time waiting for each response	The average time interval between each patient’s question and the doctor’s response	Continuous	minutes	Records
2.3 Time for consultation	The time interval between the patient’s first question and the doctor’s last response	Continuous	minutes	Records
2.4 Total times of provider’s responses	Total number of times of doctor’s responses in one encounter	Continuous	times	Records
2.5 Total words of provider’s all responses	The total number of words replied by the doctor in one encounter	Continuous	words	Records
2.6 Average words of provider’s each response	The average number of words replied by the doctor of each response in one encounter	Continuous	words	Records
3. Quality				
3.1 Adherence to checklist	Whether adherence to the case-specific checklist for urticaria in China’s national clinical guideline (2018) According to the Guideline for the Diagnosis and Treatment of Urticaria (2018) [41], twenty-three consultation items in 6 components (onset time and duration, main symptoms, following symptoms, trigger and regulation, medical record, and past history and family history) should be queried in the consultation. We defined it as the percent of items completed for clinical guideline stipulated consultation	Continuous	%	Records
3.2 Accurate diagnosis	Urticaria, not allergy, erythema annulare, popular urticaria or other diseases	Dichotomous	0: no, 1: yes	Records
3.3 Appropriate prescription	In the script, our standardized patient was designed as being treated with an antihistamine but achieved no alleviations. It is recommended by the Guideline for the Diagnosis and Treatment of Urticaria (2018) [41] that a second-line medication, such as changing the antihistamines or using other antihistamines in combination, should be prescribed. According to the Hospital Prescription Review Management Specification (Trial) [42] in China, a standardized prescription included definite medication, course duration and dosage. Therefore, we defined appropriate prescription as the doctor prescribed with a definite second-line medication with clearly defined course duration and dosage	Dichotomous	0: no, 1: yes	Records
3.4 Providing lifestyle modification advice	Whether the doctor provide lifestyle modification advice	Dichotomous	0: no, 1: yes	Records
3.5 Positive user experience	Whether SP is satisfied with the experience during the whole process of E-consults	Dichotomous	0: no, 1: yes	SP perception

### Statistical analysis

The six consultation length indicators were divided into two categories, respectively, according to the time, responses, and words, among which the time waiting for first response and each response all were divided into ≤ 60 min (1 h) and > 60 min (1 h), the time for consultation was divided into ≤ 1440 min (1 day) and > 1440 min (1 day), the total times of provider’s responses was divided into 1–2 and ≥ 3 times. To avoid quasi-complete separation, the total words of provider’s all responses was divided into ≤ 75 words and > 75 words (Statistics showed that diagnosis is always accurate when the total words of provider's all responses are above 78 in one consultation.), so the average words of provider’s each response was divided into ≤ 25 words and > 25 words.

We calculated the median (IQR) of the six indicators of consultation length according to the above classification and mean (95%CI) of the process quality and described the other four quality indicators and characteristics of E-consults services providers by the proportion. We ran ordinary least squares (OLS) regressions for process quality “adherence to checklist” and logistic regressions for the other four quality indicators to assess the association between consultation length and E-consults services quality, reporting coefficients (β) with accompanying 95% CI in OLS regressions and odds ratio (OR) with accompanying 95% CI in logistic regressions. All regressions were adjusted for key covariates (region, sex, institution, and title). We conducted statistical analyses using Stata version 15.1 (Stata Corporation, College Station, TX, USA) and considered *p* values less than 0.05 to be statistically significant (2-tailed).

## Results

### Consultation length of tele-dermatology

A total of 87 providers of tele-dermatology E-consults were included in this study. Time waiting for first response for 39(44.8%) and time waiting for each response for 25 (28.7%) of visits were ≤ 60 min, and the medians for these two indicators were 100 min (IQR: 19 ~ 243) and 157 min (IQR: 36 ~ 383), respectively. 64(73.6%) visits were completed within one day (1440 min), and the median time for consultation was 636 min (IQR: 188 ~ 1528). 59 (67.8%) of the visits had more than three times of doctor-patient interactions. Total words of provider’s all responses varied from 6 to 458 numbers of Chinese characters, with a median of 106 numbers (IQR: 56 ~ 201); average words of provider’s each response ranged from 6 to 205 numbers of Chinese characters, with a median of 32 numbers (IQR: 20 ~ 55). Table 2 shows the details.

**Table 2:** Consultation length of tele-dermatology

Variables	n (%)	median	IQR
Time waiting for first response (min)			
≤ 60 (1 h)	39(44.8)	11.00	3.00–28.00
> 60 (1 h)	48(55.2)	233.50	133.25–719.75
Time waiting for each response (min)			
≤ 60 (1 h)	25(28.7)	16.70	6.75–33.42
> 60 (1 h)	62(71.3)	243.40	138.35–461.38
Time for consultation (min)			
≤ 1440 (1 day)	64(73.6)	326.50	148.25–820.25
> 1440 (1 day)	23(26.4)	2516.00	1783.00–3019.00
Total times of provider’s responses (times)			
1–2	28(32.2)		
≥ 3	59(67.8)		
Total words of provider’s all responses (words)			
≤ 75	33(37.9)	50.00	21.50–59.50
> 75	54(62.1)	166.00	117.75–257.25
Average words of provider’s each response (words)			
≤ 25	33(37.9)	18.00	10.50–20.25
> 25	54(62.1)	48.37	33.88–76.63

### E-consults services quality of tele-dermatology

**Fig. 1:**
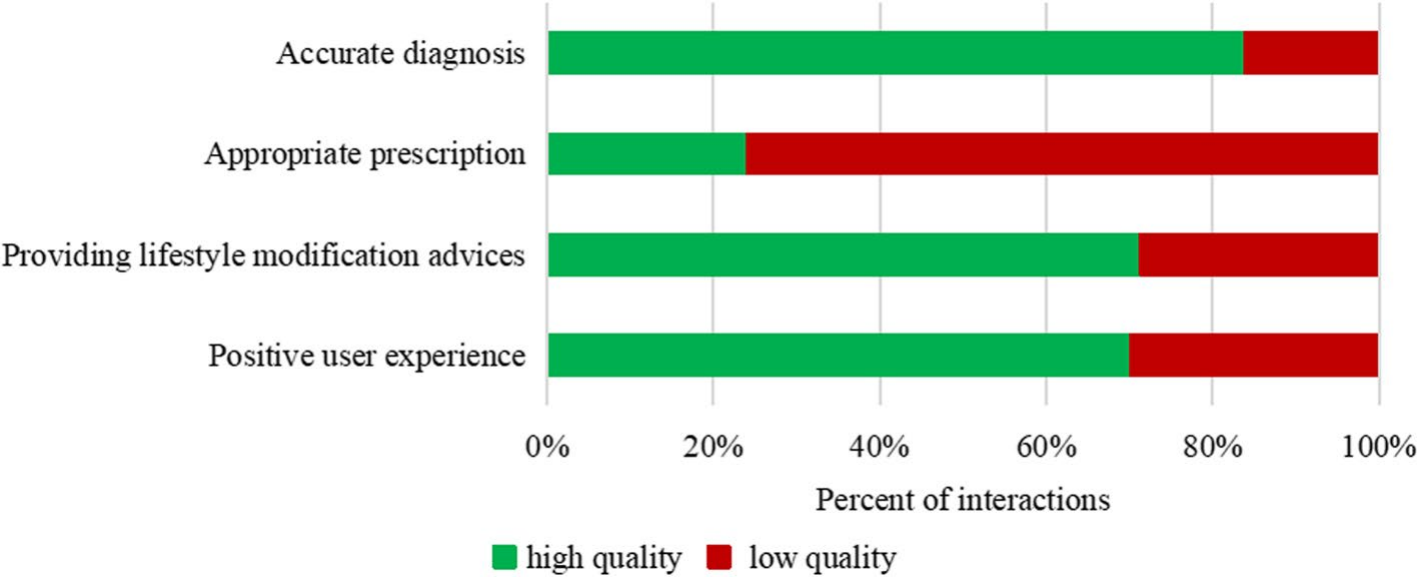
E-consults services quality of tele-dermatology

Figure 1 shows the five quality indicators results for E-consults services. The average percentage of items completed for clinical guideline stipulated consultation was only 10.84% (95% CI 9.02–12.68). 73(83.9%) were diagnosed correctly, but only 24% of the providers gave the appropriate prescription. In addition, 62 (71.3%) provided comprehensive lifestyle modification advice, and 70.1% of visits obtained satisfaction from the patient.

**Fig. 2:**
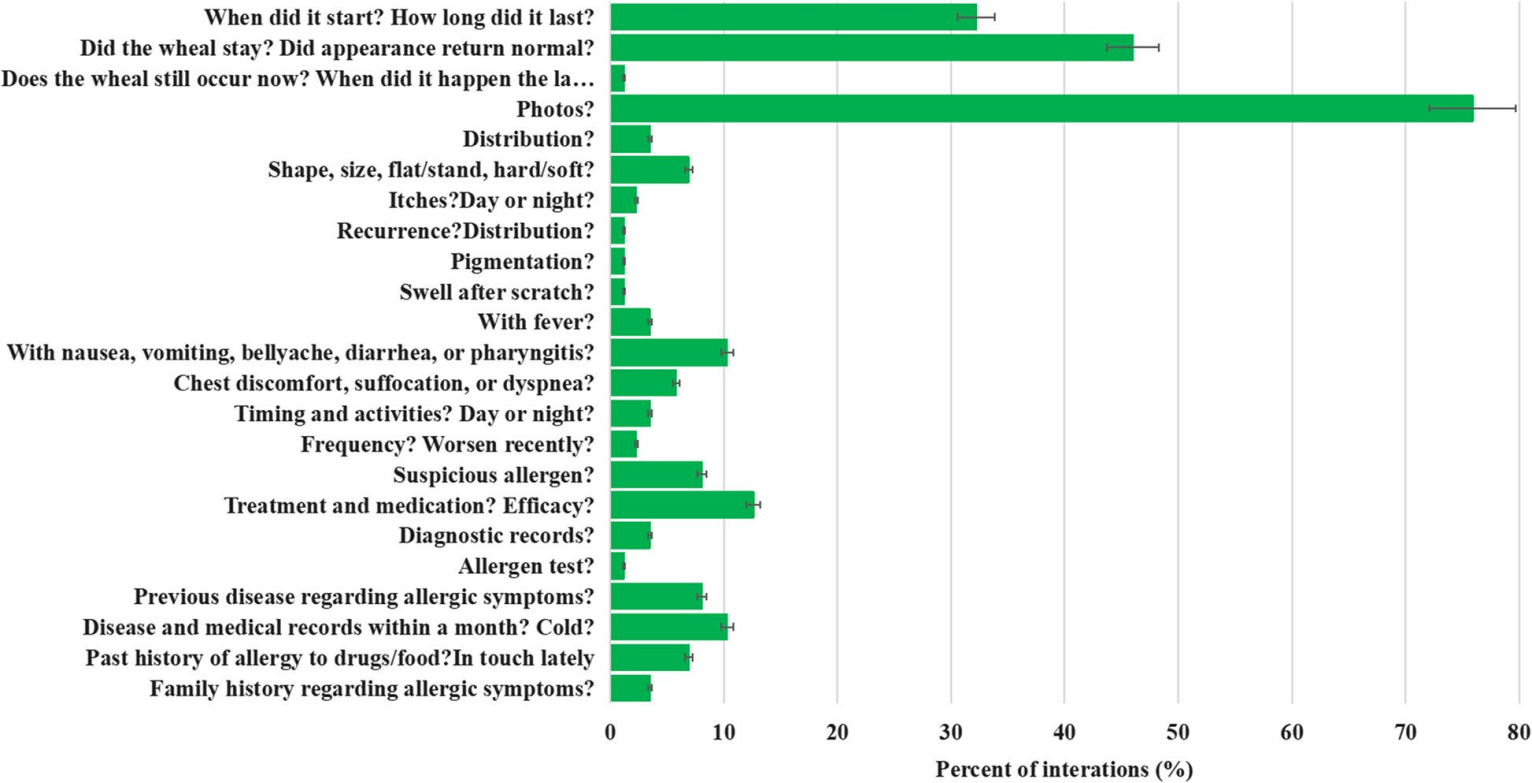
Adherence to the case-specific checklist for urticaria in China’s national clinical guideline (2018)

**Fig. 3:**
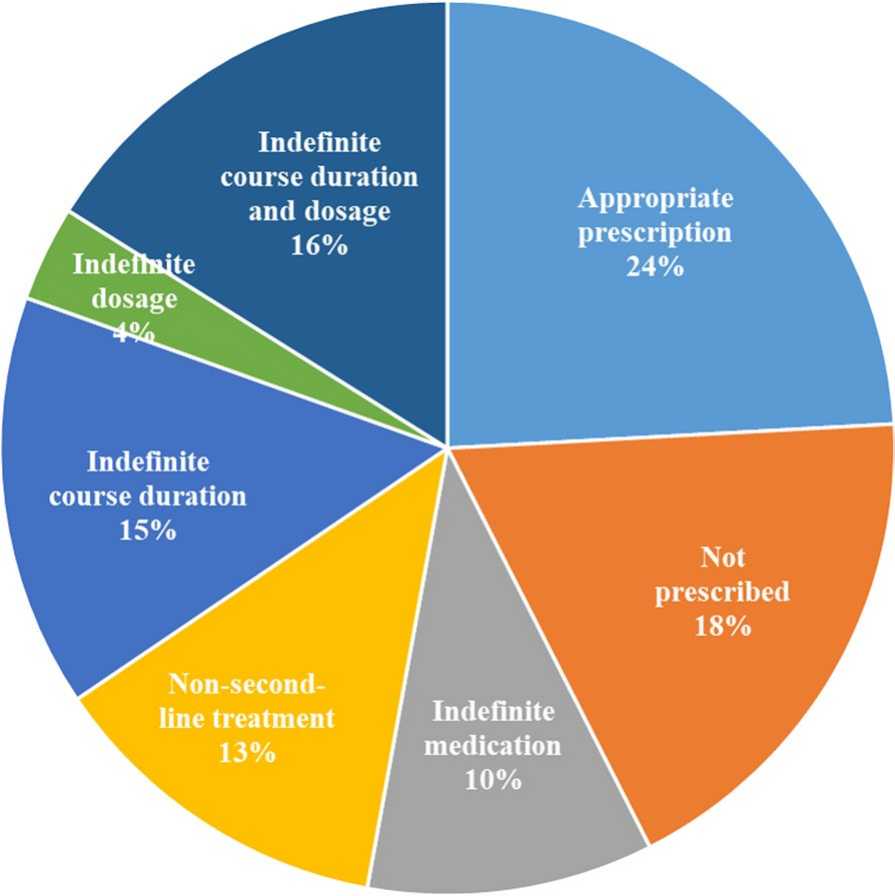
Quality of prescription

We observed poor performance in both the counseling process and the quality of prescriptions. Figure 2 presents the completion results of 23 consultation questions in 6 aspects, showing that providers asked about the photo of symptom in three-fourths of the interactions, but all other checklist items were completed in less than half of the interactions. Figure 3 shows the results for prescription quality, in which 66 providers (76%) reported inappropriate prescriptions, including not prescribed (18%), indefinite medication (10%), definite medication but non-second-line treatment (not adjusted prescriptions as recommended by the clinical guideline (13%), definite medication and second-line treatment but indefinite course duration (15%), indefinite dosage (4%), or indefinite course duration and dosage (16%).

### The relationship between consultation length and E-consult service quality

Table 3 shows the relationship between consultation length and E-consult service quality after controlling the characteristics of the healthcare. The results showed that providers who responded more promptly were likely to provide lifestyle modification advice (the adjusted ORs of time waiting for first response and each response were 9.76, 95% CI 2.47–38.58 and 4.85, 95% CI 1.16–20.36, respectively.), and obtain patient satisfaction (the adjusted ORs were 11.16, 95% CI 2.81–44.30 and 4.95, 95% CI 1.20–20.36, respectively.). The longer the time for consultation, the more likely the providers were to adhere to the case-specific checklist for urticaria in China’s national clinical guideline (2018) (adjusted β 4.20, 95% CI 0.20–8.20). Of note, the total times of provider’s responses had no effect on the prescription of appropriate medication, nor did the average words of provider's each response affect providing lifestyle advice. However, total times, total words, and average words of provider's responses had impacts on the five quality indicators. That is, providers who replied with more content were significantly more likely to adhere to the case-specific checklist, diagnose the disease accurately, give appropriate prescriptions, provide lifestyle modification advice, and obtain satisfaction from SP. Specific adjusted β/OR values are shown in Table 3.

**Table 3:** Association between consultation length and E-consults services quality

Variables	Adherence to checklist	Accurate diagnosis		Appropriate prescription		Providing lifestyle modification advice		Positive user experience	
	Adjusted β (95% CI)	%	Adjusted OR (95% CI)	%	Adjusted OR (95% CI)	%	Adjusted OR (95% CI)	%	Adjusted OR (95% CI)
Time waiting for first response (min)									
> 60 (1 h)	reference	79.2	reference	27.1	reference	56.3	reference	54.2	reference
≤ 60 (1 h)	2.15(-1.55–5.85)	89.7	2.93(0.72–11.87)	20.5	1.03(0.34–3.14)	89.7	9.76(2.47–38.58)	89.7	11.16(2.81–44.30)
Time waiting for each response (min)									
> 60 (1 h)	reference	82.3	reference	27.4	reference	64.5	reference	62.9	reference
≤ 60 (1 h)	-0.64(-4.90–3.62)	88.0	1.39(0.32–6.03)	16.0	0.95(0.24–3.66)	88.0	4.85(1.16–20.36)	88.0	4.95(1.20–20.36)
Time for consultation (min)									
≤ 1440 (1 day)	reference	81.3	reference	20.3	reference	70.3	reference	75.0	reference
> 1440 (1 day)	4.20(0.20–8.20)	91.3	2.39(0.48–11.89)	34.8	1.90(0.60–5.95)	73.9	1.15(0.38–3.52)	56.5	0.39(0.14–1.13)
Total times of provider’s responses (times)									
1–2	reference	60.7	reference	25.0	reference	32.1	reference	39.3	reference
≥ 3	6.22(2.52–9.92)	94.9	22.06(4.12–117.99)	23.7	1.24(0.39–3.94)	89.8	29.32(7.52- 114.24)	84.7	11.52(3.62–36.64)
Total words of provider’s all responses (words)									
≤ 75	reference	60.6	reference	6.1	reference	45.5	reference	39.4	reference
> 75	8.56(5.24–11.89)	98.1	49.01(4.97–483.14)	35.2	8.66(1.60–46.98)	87.0	8.95(2.81–28.43)	88.9	13.74(4.13–45.72)
Average words of provider’s each response (words)									
≤ 25	reference	66.7	reference	3.0	reference	60.6	reference	54.5	reference
> 25	6.98(3.49–10.47)	94.4	9.67(2.18–42.95)	37.0	18.87(2.13–167.03)	77.8	2.16(0.78–5.98)	79.6	3.19(1.15–8.78)

## Discussion

### Principal finding

Our study used SPs to evaluate consultation length and quality of tele-dermatology E-consults in China. To the best of our knowledge, this is the first study to explore the impact of consultation length on the quality of E-consults. Traditional measurements of consultation length in in-person visits cannot reflect the time doctors spent directly with the patient, nor can the process of doctor-patient interaction online due to the asynchronous nature of E-consults. Instead, we use six related indicators as proxies for consultation length. In our study, three main findings were present. First, providers who responded more quickly were more likely to provide lifestyle modification advice and receive satisfaction from patients without compromising the process, diagnosis, and prescribing quality. Second, providers who spent more time with patients were likely to adhere to clinical checklists. Third, we found that the total times and words of provider’s responses positively impacted the quality of tele-dermatology E-consults.

Many studies have demonstrated the clear inverse relationship between waiting time and patient satisfaction [43–45], and the same results were found in teledermatology [46]. Consistent with previous findings, our data also showed that visits with an average waiting time of ≤ 1 h were 4.85 times more likely to receive satisfaction from patients than those of > 1 h, and the same result was also found for waiting time of first response. Moreover, we found an inverse relationship between waiting time and providing lifestyle modification advice. Shorter waiting times mean that providers are more motivated to respond, and those physicians may exhibit more effective behaviors such as concern, encouragement, reassurance, empathy, and sympathy, driving them to provide lifestyle modification advice.

Furthermore, we found that visits with time for consultation of > 1 day were 4.20 times more likely to adhere to clinical checklists than those of ≤ 1 day. Under time pressure, adherence to guidelines concerning history taking was compromised; that is, physicians asked significantly fewer questions concerning presenting symptoms than the ones indicated by the guidelines [47]. A systematic review suggested that patients seeking help from a doctor who spent more time with them were more likely to have a consultation that included essential elements of care [16]. A study using standardized patients to examine the role of consultation length in delivering process quality and diagnosis quality in China also showed that the longer consultation led to better process and diagnosis quality in primary care [4]. However, these studies were set in face-to-face visits, and the measured consultation length was the time providers and patients spent during a patient's visit. Due to the asynchronous E-consults conducted in this study, time for consultation cannot represent the direct interaction between doctors and patients, which may be affected by both patient and doctor's responses.

Considering E-consults services as a new service form, no study has been found on the association between consultation length and service quality. To find alternative indicators of consultation length, we included three other indicators, namely total times of provider’s responses, total words of provider’s all responses, and average words of provider’s each response, to analyze their impact on E-consults services quality. The results showed a significant association between these three indicators and E-consults services quality among specialty care providers. Providers with more times and words of responses were significantly more likely to adhere to a clinical checklist (adequate consultation process), provide an accurate diagnosis, appropriate prescription, and lifestyle modification advice; additionally, they were more likely to obtain satisfaction from patients. Therefore, we believe that these three indicators can be used as proxies of consultation length for asynchronous online consultation. Medical consultation is always a complex temporal event [48]. The number of times and words in a provider’s responses determines how much attention has been given to patients and how much information the provider knows about the patients [16]. Thus, providers with more times and words of responses are more likely to find health problems and therefore provide higher quality care [14, 49, 50].

Our study presents excellent reasons to increase the consultation length of E-consults and has potential implications for further research and medical practice. Building on previous offline studies, we confirmed the relationship between consultation length and service quality in the setting of E-consults, which open up a massive data source for further work. In addition to standardized patients, a large amount of real-world data, i.e., online consultation data from real patients, can be used for multiple purposes and to collect other elements in the interaction between providers and patients. Of note, we found that 77% (10/13) of Internet hospital platforms have limitations on the time of doctor-patient interaction. For example, 7.7% (1/13) platforms require doctors to respond to patients three times with a maximum of 300 words; 15.4% (2/13) platforms require the asynchronous interaction between doctors and patients should be completed within 24 h. These rules may significantly limit the consultation length of E-consults and thus affect service quality. Our study underscores the importance and urgency of establishing health service rules on reliable consultation length of E-consults to ensure adequate interaction between patients and providers. To further standardize online medical care, promote its healthy development, and ensure medical quality and safety, the National Health Commission of People’s Republic of China issued the "Rules on Supervision of Internet Diagnosis and Treatment (Consultative Draft)" in October 2021. In this regard, we suggest that affordable and reliable consultation length, response times and words of E-consults is required to clarify to continuously adapt to the potential challenges of digital health.

### Limitations

This study has several limitations. First, we only investigated tele-dermatology E-consults, which results in our findings may not be generalizable to wider samples of the healthcare system due to the enormous variability among different diseases. Further research is underway on other conditions, including chronic diseases such as diabetes and mental disorder such as depressive disorders. Second, our survey was set in regions with a better development level of DTC telemedicine in China, and the research conclusion cannot represent the overall situation in China. Our follow-up investigation will further extend to other regions of China. Third, we did not analyze the impact of patient behavior on outcomes, mainly because we used standardized patients trained to visit the physician online strictly according to a standard script. However, real-world data may be affected by the behavior of real patients, and patient characteristics and response should be included as control variables.

## Conclusions

In face-to-face visits, longer consultations were associated with a range of better patient outcomes [51]; Our results indicated that in asynchronous E-consults, more times and words of provider’s responses were associated with the process, diagnosis, prescription quality, lifestyle modification advice, and patient satisfaction. The total times and words of the provider's responses can be used as proxies of consultation length of E-consults, which also might become a proxy measure of the quality of care and influence the delivery of health care. Future research should consider the benefits of longer consultations across a wider range of practices.

## Supplementary Information

**Additional file 1:** Selection conditions of SP cases and characteristics of urticaria.

**Additional file 2:** Examples of specific consultations in English and Chinese.

**Additional file 3:** Characteristics of the tele-dermatology E-consults services providers.

## Data Availability

The datasets generated during and/or analysed during the current study are available from the corresponding author on reasonable request.

## Abbreviations

SPs: Standardized Patients
DTC: Direct-to-consumer
