# Supplementary material for: Investigating the relationship between consultation length and quality of tele-dermatology E-consults in China: a cross-sectional standardized patient study
Source: BMC Health Serv Res. 2022 Sep 22;22:1187. doi: 10.1186/s12913-022-08566-2 (PMC9493166; doi:10.1186/s12913-022-08566-2)
Supplement: Supplementary file 1 — Additional file 1: Selection conditions of SP cases and characteristics of urticaria. [file 12913_2022_8566_MOESM1_ESM.docx]

**Additional file 1**

Table 1 Selection conditions of SP cases and characteristics of urticaria

| Conditions | Characteristics of urticaria |
| --- | --- |
| Technical feasibility |  |
| Can a trained SP portray the case? | The wheals will go away naturally, without raising a doctor's suspicions. Besides, the symptoms of urticaria are simple and do not cause strong physical discomfort, so SP is easy to play. |
| Do national or international guidelines exist for correct management or treatment? | Yes. Urticaria in China’s national clinical guideline (2018). |
| Can expected management be performed within one visit? | Yes. No follow-up visit is required. |
| Ethical acceptability |  |
| Does the case choice minimize potential harm to fieldworkers? | No potential harm to fieldworkers. |
| Does the case require the involvement of children? | No. |
| Appropriateness to context and research question |  |
| Is the case appropriate to the study objective? | Yes. Dermatosis is one of the main diseases in Internet diagnosis and treatment in China. |
| Do stakeholders agree the case is a ‘fair test’? | They all agree. |
| Is the case applicable to all health facilities and regions in the study? | Yes, the research team has screened hospitals and doctors that meet the requirements of the study. |
| Does the case represent a public health concern? | In the past 30 years, the incidence of allergic diseases has increased at least threefold and has reached as high as 20%. The incidence of urticaria in China has also continued to rise, with 9% to 20% of people ever suffering from urticaria. |
| Does the case match local epidemiology? | Dermatosis is one of the earliest telemedicine diseases because of its visual diagnosis. Dermatology consultation volume ranked top 5 in Beijing tertiary hospitals, and dermatology consultation volume increased significantly after the epidemic. |
