# Supplementary material for: Investigating the relationship between consultation length and quality of tele-dermatology E-consults in China: a cross-sectional standardized patient study
Source: BMC Health Serv Res. 2022 Sep 22;22:1187. doi: 10.1186/s12913-022-08566-2 (PMC9493166; doi:10.1186/s12913-022-08566-2)
Supplement: Supplementary file 2 — Additional file 2: Examples of specific consultations in English and Chinese. [file 12913_2022_8566_MOESM2_ESM.docx]

**Additional file 2**

The following are examples of specific consultations in English and Chinese:

**Case 1**

07/05/2021 15:20 SP: Doctor, there have been wheals all over my body with itch. Could you please figure this out for me?

07/05/2021 15:20 SP：医生您好，我这段时间全身皮肤长红色大疙瘩，很痒，想咨询您看看怎么回事，怎么治疗?

07/05/2021 19:08 D: (1) No photo? (2)How long have you felt sick? A few days? A few weeks? A few months? (3) The wheals always exist? Or with recurrence? (4) What is the regulation and trigger? Cold and hot? Scratching? Stressed out? Certain food stimuli? Please fill in the above information, the more detailed, the better.

07/05/2021 19:08 D：（1）无照片?（2）病史多久，几天？几周？数月？（3）这些红色大包，是一直存在，还是时起时消？（4）发病规律有哪些，和遇冷遇热有关？还是和抓挠有关?还是和精神压力大或者某些食物刺激有关？请补充以上信息，越详细越好。

07/05/2021 19:09 SP: (1) Photo. (2) Almost a month. (3) With recurrence. (4) It is usually found when I have a rest after dinner or just when I get up. Nothing else.

07/05/2021 19:09 SP：（1）提供图片。（2）病史差不多一个月了。（3）时起时消。（3）一般都是吃晚饭后休息的时候或者刚起床的时候发现起包。我没发现和什么有关系。

07/05/2021 19:28 D: Typical chronic urticaria. Please think carefully. Do you have any of these diseases: 1. Hyperthyroidism or hypothyroidism? 2. Familial inherited diabetes? 3. Other chronic diseases, such as chronic enteritis or gastric ulcer? If you do not have the disease mentioned above, you can concentrate on treating this spontaneous skin disease. If you have the above diseases, please specialize in treatment.

Anti-allergy treatment is as follows：(1) one Loratadine tablet in the morning. (2) One Ebastine tablet before going to bed. (3) Take 0.6-1g of Vitamin C every day. All of the above medications can be taken before or after meals. Adhere to the treatment for a month and then have a follow-up visit; the dosage is usually reduced successfully. All of the above drugs are safe and can be taken for a long time.

07/05/2021 19:28 D：是典型的慢性荨麻疹症状，请仔细想一下，平时有没有这几种疾病：1、甲亢或者甲减？2、家族遗传性糖尿病？3、其他的一些慢性疾病，比如说慢性肠炎或者胃溃疡？如果平时很健康，没有上述疾病的话，就专心治这个自发性皮肤病就可以了。如果有以上疾病，请专科专治。

抗过敏治疗如下：（1）早晨一片氯雷他定片。（2）睡前一片依巴斯汀片。（3）每天补充0.6-1g维生素C。以上药物均和饭前饭后无关。坚持治疗一个月后再复诊，一般能顺利减药量。以上药物都非常安全，均可长期服用。

07/05/2021 19:39 SP: I don't have any of these diseases. Is there anything else I should be aware of?

07/05/2021 19:39 SP：1、2、3都没有。那我平时有什么需要注意的吗?

07/05/2021 23:23 D: (1) You are a woman with no alcohol and tobacco problems. It would help if you tried to eat home-cooked meals, fewer restaurants, and try not to eat packaged food (with food additives). (2) Do not eat such frozen meatballs, crab sticks, or other convenient food that cannot see the original ingredients. (3)Both meat and vegetables can be eaten. If you find a fruit or seafood that can cause wheals quickly, avoid it. (4) Pillow core bedding should often be directly basked in by the sun. Always ventilate the room. The old ash must be cleaned. Wish you health!

07/05/2021 23:23 D：（1）你是女性，没有烟酒的困扰，平时尽量吃自己家里做的饭，少下馆子，尽量不吃袋装食品（食品添加剂）。（2）不摄入类似速冻丸子，鱼豆腐，蟹棒之类看不清原始食材的方便食品。（3）荤素不忌，该吃照吃。万一发现某种水果或者海鲜，吃完几分钟或者几小时内出现荨麻疹皮疹，就尽量避免。（4）枕芯、被褥要经常放太阳下直晒，室内通风，陈年老灰务必打扫干净。祝健康。

END

**Case 2**

07/05/2021 18:03 SP: Doctor, there have been wheals all over my body with itch. Could you please figure this out for me?

07/05/2021 18:03 SP：医生您好，我这段时间全身皮肤长红色大疙瘩，很痒，想咨询您看看怎么回事，怎么治疗?

07/05/2021 20:28 D: Did its appearance return to normal within 48 hours?

07/05/2021 20:28 D：红色疙瘩48小时内能消退变平吗?

07/05/2021 20:51 SP: Yes.

07/05/2021 20:51 SP：嗯，差不多是的

07/05/2021 22:47 D: It's probably urticaria. Take some anti-allergy medication. Don't scratch, and you can use some calamine lotion. You should go to the hospital in three days if it does not work. You should be hospitalized immediately for observation if you have a sore throat, watery stool, or severe abdominal pain.

07/05/2021 22:47 D：可能是荨麻疹，吃点抗过敏药物，不要抓挠，可以抹点炉甘石。三天解决不好应该能医院看看。如果嗓子很疼，或水样便，或腹痛剧烈，应当马上住院观察。

08/05/2021 09:16 SP: Is there anything else I should be aware of?

08/05/2021 09:16 SP：我平时还有什么需要注意的吗?

The doctor hadn’t answered until the end of the visit.

直到问诊结束医生未回复。

**Case 3**

17/05/2021 07:55 SP: Doctor, there have been wheals all over my body with itch. Could you please figure this out for me?

17/05/2021 07:55 SP：医生您好，我这段时间全身皮肤长红色大疙瘩，很痒，想咨询您看看怎么回事，怎么治疗

17/05/2021 22:48 D: You'd better go to the hospital to see a doctor. If you have abdominal discomfort, chest tightness, or other systemic reactions, you should visit the hospital in time for examination and treatment.

17/05/2021 22:48 D：最好来医院看一下，如果有腹部不适、胸闷等全身反应，及时到医院检查治疗。

18/05/2021 08:52 SP: Doctor, what could it be?

18/05/2021 08:52 SP：医生，那我这可能是什么病呢？

The doctor hadn’t answered until the end of the visit.

直到问诊结束医生未回复。
