# Supplementary material for: Investigating the relationship between consultation length and quality of tele-dermatology E-consults in China: a cross-sectional standardized patient study
Source: BMC Health Serv Res. 2022 Sep 22;22:1187. doi: 10.1186/s12913-022-08566-2 (PMC9493166; doi:10.1186/s12913-022-08566-2)
Supplement: Supplementary file 3 — Additional file 3: Characteristics of the tele-dermatology E-consults services providers. [file 12913_2022_8566_MOESM3_ESM.docx]

**Additional file 3**

Table S1 Characteristics of the tele-dermatology E-consults services providers

| Characteristics | n | % |
| --- | --- | --- |
| Region (n=87) |  |  |
| Beijing | 35 | 40.2 |
| Hangzhou | 52 | 59.8 |
| Sex (n=85)^a^ |  |  |
| Male | 29 | 34.1 |
| Female | 56 | 65.9 |
| Institution (n=87) |  |  |
| Traditional Chinese medicine hospital | 30 | 34.5 |
| General hospital | 57 | 65.5 |
| Title of the doctor (n=84)^b^ |  |  |
| Senior | 34 | 40.5 |
| Junior | 50 | 59.5 |

^a^ Two doctors have missing values in sex.

^b^ Three doctors have missing values in title.
